# Diphyllobothriasis, Brazil

**DOI:** 10.3201/eid1110.050377

**Published:** 2005-10

**Authors:** Jorge Luiz Mello Sampaio, Victor Piana de Andrade, Maria da Conceição Lucas, Liang Fung, Sandra Maria B. Gagliardi, Sandra Rosalem P. Santos, Caio Marcio Figueiredo Mendes, Maria Bernadete de Paula Eduardo, Terry Dick

**Affiliations:** * Fleury Research Institute, São Paulo, Brazil; † São Paulo State Department of Health, São Paulo, Brazil; ‡ University of Manitoba, Winnipeg, Manitoba, Canada

**Keywords:** Diphyllobothrium latum, diphyllobothriasis, Brazil, dispatch

## Abstract

Cases of human diphyllobothriasis have been reported worldwide. Only 1 case in Brazil was diagnosed by our institution from January 1998 to December 2003. By comparison, 18 cases were diagnosed from March 2004 to January 2005. All patients who became infected ate raw fish in sushi or sashimi.

Diphyllobothriasis is an intestinal parasitosis acquired by eating raw or partially cooked fish containing *Diphyllobothrium* spp. plerocercoids. Most persons are asymptomatic, but diarrhea, abdominal pain, or discomfort occurs in <22% of infections. Prolonged or heavy *Diphyllobothrium latum* infection may cause megaloblastic anemia due to parasite-mediated dissociation of the vitamin B_12_–intrinsic factor complex within the gut lumen, making B_12_ unavailable to the host (*1*).

Human diphyllobothriasis has been reported in Europe, Asia, North America, and South America. South American has reported cases from Peru, Chile, and Argentina, but not Brazil (*2**–**7*). South American diphyllobothriasis is an ancient disease; *D. pacificum* eggs were found in coprolites, 4,000- to 5,000-year-old Chinchorro Chilean mummies (*8*).

Four recognized species, *D. latum*, *D. pacificum*, *D. klebanovskii*, and *D. nihonkaiense*, infect humans; many species infect fish-eating birds, dogs, foxes, and bears (*2**,**5*). Species identification is relevant because *D. pacificum* infects only saltwater fish. *D. latum* infects only freshwater fish or species that spend part of their life in fresh water. Only *D. latum* and *D. pacificum* have been found in humans in South America; other species of *Diphyllobothrium* have been found in freshwater fish from Chile and Argentina (*9**,**10*).

## The Study

Since diphyllobothriasis was a rare disease in Brazil, 5 cases diagnosed from March to August 2004 were of interest. At our São Paulo institution, ≈36,000 stool specimens are examined for ova and parasites annually. Since 1998, no changes in personnel or protocols used for stool examination have occurred.

A database was searched for the period from January 1998 to December 2003 to determine the number of our patients diagnosed with *Diphyllobothrium* infection. From September 2004 to January 2005, stool specimens of patients who ate raw fish were examined to determine the prevalence of diphyllobothriasis. Patients >15 years of age were asked if they had eaten raw fish in the past 2 months. All patients, except those with *Diphyllobothrium* eggs in their stools, were asked if they had been sick, if they had eaten raw fish, the species of fish eaten, and if they had traveled outside Brazil in the last 5 years. When available, hemoglobin and mean corpuscular volume samples were evaluated to exclude megaloblastic anemia.

Ten eggs were randomly sampled from *Diphyllobothrium* spp.–positive stool specimens from 4 randomly chosen patients; the length and width of the eggs were recorded. Strobilas obtained from 2 specimens were evaluated by scanning electronic microscopic studies (*11*). Fragments of strobila were fixed in 10% formalin, embedded in paraffin, sectioned, and stained with hematoxylin-eosin. Uterine morphology was evaluated in fresh preparations of proglottids.

The database search found 1 case of diphyllobothriasis. Comparatively, 5 cases were diagnosed from March to August 2004. To determine the prevalence of diphyllobothriasis, we added a standard question about eating raw fish to our admission protocol from September 2004 to January 2005. During this period, we examined fecal specimens of 8,463 patients >15 years of age. Among those patients, 623 refused to admit eating raw fish, 5,335 denied eating raw fish, and 2,505 stated they did eat raw fish. Thirteen cases, 5.19/1,000, of diphyllobothriasis were found in the patients who ate raw fish. The infected patients were from 16 to 59 years of age with a mean age of 33 years. None of the patients who denied eating raw fish had *Diphyllobothrium* eggs in their specimens. The most frequently reported symptoms were abdominal discomfort and intermittent diarrhea (83.3%). Twenty-two percent of the patients eliminated the parasite; 16.7% were asymptomatic. Mean corpuscular volume was 81.l–93.9 fL and hemoglobin was 12.2–16.8g/dL in 8 patients. All values were within the normal range. Seven of the 18 patients had not traveled outside Brazil in the last 5 years; 2 of them had never traveled outside Brazil. Eggs observed in stool samples had the characteristic shape observed in *Diphyllobothrium* spp. (Figure, A). The average length was 64–71 μm, and the average width was 48–51 μm.

**Figure:**
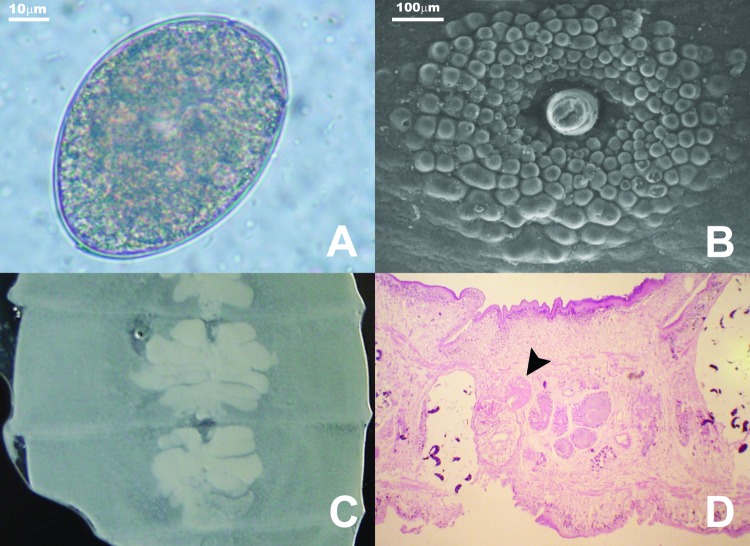
A) Diphyllobothrium latum egg. Note opercular constriction. B) Genital papillae of mature proglottids as seen under scanning electron microscope. C) Uterine loops of gravid proglottids in fresh preparation. D) Sagittal section of the genital pore region stained with hematoxylin-eosin. Note seminal vesicle (arrowhead) situated dorsocaudal to the cirrus sac (magnification 100×).

Of the 4 adult worm specimens (strobila fragments) available for analysis, the largest fragment was 900 mm long by 7 mm wide with an average proglottid length of 2.5 mm. Scanning electronic microscopic studies showed circular and conspicuous genital papillae at the upper third of proglottids (Figure, B). Uteri from gravid proglottids formed a rosette with 4–5 loops (Figure, C). Sagittal sections of adult worms showed the cirrus sac lying horizontally with the seminal vesicle lying dorsocaudal to it. A marked constriction between segments was noted (Figure, D).

The presence of genital papillae showed that the specimens from Brazilian patients were similar to those in published reports for *D. latum* (*11*). Sagittal sections of the genital pore region of worms in Brazilian patients showed the cirrus sac lying horizontally with the seminal vesicle lying dorsocaudal to it, as described by Dick et al. (*12*). Our findings exclude *D. pacificum* since uteri from gravid proglottid of worms in Brazilian patients formed a rosette, which was not observed in *D. pacificum*. The largest reported *D. pacificum* eggs (40 μm wide and 60 μm long) (*13*) were smaller than the smallest eggs (48 μm wide and 64 μm long) in our study. Although egg dimension can not be used as a single criterion for species identification, the values for *D. latum* recorded in our study agree with Andersen et al. (*14*).

Based on the genital papillae, position of the cirrus sac, shape of the uterus, and egg dimensions, we tentatively identify the specimens as *D. latum* and implicate this species as the source of human infection in São Paulo. All infected patients ate raw fresh Atlantic salmon and some ate a local fish, *Centropomus undecimalis*, in sushi or sashimi. *C. undecimalis* has not been reported as a *D. latum* host, but it is a saltwater fish that spends part of its life in fresh water.

## Conclusions

Since the Brazilian climate prevents Atlantic salmon farming, it is imported from Chile, where diphyllobothriasis is endemic in some regions (*15*). The 18 infections we describe were acquired in Brazil because 2 patients had never traveled outside the country. Imported salmon may be the source of *D. latum* plerocercoids infections; another possibility is that the life cycle of *D. latum* is established in São Paulo coastal waters and rivers. If so, *C. undecimalis*, used in sushi and sashimi, could be a source of infection.

Epidemiologic investigations are being conducted in São Paulo that will identify the source of *D. latum* plerocercoids and help implement educational and sanitary measures and prevent diphyllobothriasis from becoming endemic in Brazil.

## References

[1] King CH. Cestodes. In: Mandell GL, Bennett JE, Dolin R, editors. Principles and practice of infectious diseases. 6th ed. Philadelphia: Elsevier Churchill Livingstone, Inc.; 2005. p. 3285–93.

[2] Dick TA, Nelson PA, Choudhury A. Diphyllobothriasis: update on human cases, foci, patterns and sources of human infections and future considerations. Southeast Asian J Trop Med Public Health. 2001;32(Suppl 2):59–76. 12041607

[3] Dupouy-Camet J, Peduzzi R. Current situation of human diphyllobothriasis in Europe. Euro Surveill. 2004;9:31–4. 15208471

[4] Semenas L, Kreiter A, Urbanski J. New cases of human diphyllobothriasis in Patagonia, Argentine. Rev Saude Publica. 2001;35:214–6. 10.1590/S0034-89102001000200017 11359210

[5] Sagua H, Neira I, Araya J, González J. Nuevos casos de infección humana por *Diphyllobothrium pacificum* (Nybelin, 1931) Margolis, 1956 en Chile y su probable relación con el fenómeno de El Niño, 1975–2000. Bol Chil Parasitol. 2001;56:22–5. 10.4067/S0365-94022001000100006 12058668

[6] Flores JM, Vidaurre MT, Rivera ML, Rosales MC. *Diphyllobothrium pacificum* en niños del Peru. Diagnóstico. 2002;41:161–4.

[7] Lee KW, Suhk HC, Pai KS, Shin HJ, Jung SY, Han ET, *Diphyllobothrium latum* infection after eating domestic salmon flesh. Korean J Parasitol. 2001;39:319–21. 10.3347/kjp.2001.39.4.319 11775333 PMC2721218

[8] Reinhard K, Urban O. Diagnosing ancient diphyllobothriasis from Chinchorro mummies. Mem Inst Oswaldo Cruz. 2003;98(Suppl 1):191–3. 10.1590/S0074-02762003000900028 12687781

[9] Torres P, Aedo E, Figueroa L, Siegmund I, Silva R, Navarrete N, Infección por helmintos parásitos en salmón coho, *Oncorhynchus kisutch*, durante su retorno al río Simpson, Chile. Bol Chil Parasitol. 2000;55:31–5. 10.4067/S0365-94022000000100009 11757416

[10] Revenga JE. *Diphyllobothrium dendriticum* and *Diphyllobothrium latum* in fishes from southern Argentina: association, abundance, distribution, pathological effects, and risk of human infection. J Parasitol. 1993;79:379–83. 10.2307/3283573 8501594

[11] Yamane Y, Bylund G, Abe K, Osaki Y, Okamoto T. Scanning electron microscopic study of four *Diphyllobothrium* species. Parasitol Res. 1989;75:238–44. 10.1007/BF00931282 2710776

[12] Dick TA, Poole BC. Identification of *Diphyllobothrium dendriticum* and *Diphyllobothrium latum* from some freshwater fishes of central Canada. Can J Zool. 1985;63:196–201. 10.1139/z85-030

[13] Baer JG, Miranda HC, Fernandez RW, Medina JT. Human diphyllobothriasis in Peru. Z Parasitenkd. 1967;28:277–89. 10.1007/BF00260267 5628217

[14] Andersen K, Halvorsen O. Egg size and form as a taxonomic criteria in *Diphyllobothrium* (Cestoda, Pseudophyllidea). Parasitology. 1978;76:229–40. 10.1017/S0031182000047818 652389

[15] Torres P, Gesche W, Montefusco A, Miranda JC, Dietz P, Huijse R. Diphyllobothriasis humana y en peces del lago Riñihue, Chile: efecto de la actividad educativa, distribución estacional y relación con sexo, talla y dieta de los peces. Archivos de Medicina Veterinaria. 1998;30:31–45. 10.4067/S0301-732X1998000100004

